# Bovine Interferon-Tau Activates Type I interferon-Associated Janus-signal Transducer in HPV16-positive Tumor Cell

**DOI:** 10.7150/jca.33527

**Published:** 2020-06-01

**Authors:** Geny Fierros-Zárate, Clarita Olvera, Gustavo Salazar-Guerrero, Ausencio Morales-Ortega, Fernando Reyna, Eva Hernández-Márquez, Eduardo Guzmán-Olea, Ana I. Burguete-García, Vicente Madrid-Marina, Oscar Peralta-Zaragoza, Marilú Chávez-Castillo, Víctor Hugo Bermúdez-Morales

**Affiliations:** 1 Centro de Investigación sobre Enfermedades Infecciosas, Instituto Nacional de Salud Pública, Cuernavaca Morelos, México.; 2 Departamento de Ingeniería Celular y Biocatálisis, Instituto de Biotecnología, Universidad Nacional Autónoma de México, Cuernavaca Morelos, México.; 3 Catedrático Consejo Nacional de Ciencia y Tecnología (CONACYT). Instituto de Ciencias de la Salud (ICSA), Universidad Autónoma del Estado de Hidalgo (UAEH), México.; 4 Universidad Tecnológica Emiliano Zapata de Morelos, UTEZ.

**Keywords:** IFN-τ, type 1 interferon, signal transducer, BMK-16/myc, tumor cells

## Abstract

The mechanisms of signal transduction by interferon-tau (IFN-τ) are widely known during the gestation of ruminants. In trophoblast cells, IFN-τ involves the activation of the JAK-STAT pathway, and it can have effects on other cell types, such as tumor cells. Here we report that the HPV16-positive BMK-16/myc cell treated with ovine IFN-τ, results in the activation of the canonical JAK-STAT and non-canonical JAK-STAT pathway. The MAPK signaling pathway was activated, we detected the proteins MEK1, MEK2, Raf1, STAT3, STA4, STAT5 and STAT6. Moreover, IFN-τ induced the expression of MHC Class I, MX and IP10 in the tumor cells and this response may be associated with the viral replication and with the anti-proliferative and the immunoregulatory effects of IFN-τ.

## Introduction

Interferon-tau (IFN-τ) is a type 1 interferon produced by the mononuclear trophectoderm. It signals the process of maternal recognition of pregnancy in ruminants, but it is now known to have a plethora of physiological functions in the mammalian utero 1. Despite the fact that it has similar properties to those displayed by classical type I IFNs and that it binds to the same receptor (IFNAR), it is remarkably less toxic, even at high concentrations; it is able to cross species barriers 2; it is not virally inducible, and its biological functions are unrelated to pathogenesis 3.

Like other classical type I IFNs, IFN-τ signals use the same receptor, associating two membrane proteins, IFNAR1 (α-subunit) and IFNAR2 (β-subunit) 4. It has also been shown that bovine IFN-τ and bovine IFN-α bind to the same receptor with similar dissociation constants 5. The interaction of the IFN-τ receptor results in the activation of canonical and non-canonical signaling pathways in endometrial cells (EC). The canonical pathway involves the activation of tyrosine kinases (JAK kinase), leading to the phosphorylation and the dimerization of the STAT transcription factors (JAK-STAT pathway), which results in the induction of an IFN-I-stimulated response element (ISRE) leading to the activation of the transcription of interferon-stimulated genes (ISGS) 6,7. Recent evidence indicates that the type-I IFN signaling pathway could be establishing non-canonical pathways side-by-side with the canonical ones, including IFN-τ 6. However, the non-canonical pathway mediated by IFN-τ and interferon-stimulated genes has not been entirely elucidated, but it has been speculated that IFN-τ must use a STA1-independent signaling pathway to regulate gene expression in the endometrial luminal epithelium of the ovine uterus 8. It has been described that other classical type I IFNs can cooperate and coordinate with multiple distinct signaling cascades, including the mitogen-activated protein kinase (MAPK) cascade and the phosphatidylinositol 3-kinase (PI3K) cascade, to generate a response 9.

It has been widely determined that during the pregnancy recognition process in the uterine endometrium, IFN-τ promotes the expression of many ISGS in a cell-type specific manner through an intracellular signal transduction system involving Stats and IFN regulatory factors (IRFs) 10,11. These mechanisms appear to be very finely regulated during pregnancy, but the way in which IFN-τ acts against different cells (non-endometrial stromal cells, peripheral blood lymphocytes (PBL), tumor cells, virus-infected cell, etc.) has not yet been determined. Recently, we reported that bovine IFN-τ induces a greater antiproliferative effect and an apoptosis in human (SiHa) and murine (BMK-16/myc) cells transformed with human papillomavirus genotype 16. Furthermore, IFN-τ represses the E6 and E7 HPV 16 oncogenes, and it displays tumor inhibition. Moreover, both of the cell-lines mentioned expressed the type-I interferon receptor (IFNAR) 12. These findings show that the HPV 16-transformed epithelial cells respond to IFN-τ, and that it can have different alternative pathways to generate these biological effects, which have a therapeutic potential.

In our work, we hypothesize that IFN-τ activates the canonical signaling pathways (JAK-STAT pathway) in the HPV 16-transformed epithelial cells, and that it favors the transcription of the ISGS (MCH class I, MX, IP10). Our results indicate that the stimulation of the transformed epithelial cells with IFN-τ results in an activation of the classical JAK-STAT pathway. Moreover, both phosphorylated and non-phosphorylated forms of the STAT1, JAK1, and TYK2 proteins were detected in the transformed epithelial cells activate by IFN-τ, along with alternative pathways (MAPK cascade, MEK1, MEK2, Raf1, STAT3, STA4, STAT5 and STAT6). We also detected the IFN-τ-stimulated expression of the MHC Class I, MX and IP10 genes. These results have not been reported with respect to HPV16-transformed tumor cells, and the activation of IFN-τ has not been described.

## Materials and Methods

### Cell lines and Chemicals

A murine HPV 16-transformed BMK-16/myc cell line, which produces tumors in immunocompetent mice 13,14, was established by co-transformation of baby Balb/c kidney cells with the c-myc gene and the HPV 16 genome. The cell line was cultured in Dulbecco's Modified Eagles Medium (DMEM) supplemented with 10% of fetal bovine serum (FBS) (Gibco, ThermoFisher Scientific, Waitham, MA, USA), ampicillin, streptomycin and amphotericin (Caisson), at 37ºC in 95% humidity with 5% CO_2_. IFN-τ ovine recombinant protein was obtained through Prospec, Rehovot Israel. Luna Universal Probe one-step RT-qPCR kit (New England Biolabs, Ipswich, MA, USA) primers were used to detect MHC Class I, MX, and IP10 was synthetized at the Biotechnology Institute in Mexico.

### JAK-STAT Profiling in BMK-16/myc Cells Treated with Ovine IFN-τ

The JAK-STAT-protein profiling was performed using the Jak/Stat Phospho Antibody Array (PJS042) and the antibody Array Assay Kit (KAS02), designed and manufactured by Full Moon Biosystems (Sunnyvale, CA, USA). The proteins microarray analysis was carried out according to the manufacturer's instructions. Briefly, 5X10^6^ BMK-16/myc cells were cultivated on cell culture flasks and were treated with 100 ng/ml of ovine IFN-τ during 15 min; a non-treated cell culture was used as control. After the cells were harvested using a scraper and lysed (with Lysis beads and buffer), the clarified-protein concentrations were determined using a BCA protein assay kit (Pierce, Rockford, Ill, USA). One hundred micrograms of cell lysate was labeled with biotin in DMF (10 μg/μl) (N,N-Dimethylformamide), and biotin-labeled were diluted 1:20 in a coupling solution before applying them to the array for conjugations. The antibody microarray was blocked before the incubation with the biotin-labeled cell lysates at room temperature (RT) during 4 hrs. Then, the slides were washed; subsequently, 60 μ of Cy3-streptavidin in detection buffer (0.5mg/ml) were added, and the mix was incubated for 20 min at RT. After the slides were washed with Milli-Q grade water, they were dried by centrifugation before the fluorescence detection.

### Data Acquisition and Analysis of Array Images

Acquisition and quantification of array images was performed in a GenePix 4100A with its accompanying software GenePix from Molecular Divides. All images were captured using a 10-μm resolution. For each spot, the Cy3 density mean value and the background mean value were calculated with software ArrarPro Anayzer from Media Cibernetics. The results from quadruplicate samples were averaged, and the *GAPDH* density mean values were used to normalize the value of the JAK-STAT-protein detection. This was compared with the control (BMK-16/myc cells without treatment).

### Detection of IFN-τ-Stimulated Genes by Real Time RT-PCR

BMK-16/myc cells were cultivated and were treated with 0 and 100 ng/ml of ovine IFN-τ for 48 hr. Total RNA was isolated using Trizol Reagent (Invitrogen) according to the manufacturer's instructions. The detection of mRNA MHC Class I, MX and IP10 was carried out using the ViiA 7 Real-Time PCR system (Thermo Fisher Scientific) with Luna Universal Probe One-Step RT-PCR Kit (New England BioLabs). Briefly, the RT-PCR reaction included 5 μL One-Step reaction mix 2X, 0.5 μL Luna warmstart RT enzyme mix (20X), 10 UM of each primer, a 10 uM probe, Nuclease-free water, and 100 ng of RNA sample in a 10 μL final reaction volume. The thermocycler conditions were as follows: Stage 1: 55ºC for 10 min; Stage 2: 95ºC for 1 min; Stage 3: 95ºC for 10 sec, 60ºC for 1 min. This was repeated for 40 cycles. Assays were carried out in triplicate and prepared for each target mRNA and an internal control gene (GAPDH). RT-qPCR primers and an appropriate probe were chosen by a Universal Probe Library (UPL) assay design center web service (Roche Applied Science). For each gene, the chosen RT-qPCR assay was the most highly ranked by the design software. The primer sequence for MHC class I was a forward 5´-CTCAGCTCCGCCTTGAAT-3´ and Reverse 5´-TCACTGGGAGAGGTACACT CAG-3´ and (FAM) and TaqMan probe No. 74. For IP10, the sequences were 5´-TCTCACTGGC CCGTCATC-3´ and Reverse 5´-GCTGCCGTCATTTTCTGC-3´and TaqMan probe No. 3. For MX2, 5´-GCTTTCCCAGGACCATCC-3´and Reverse 5´-GCTTTCCCAGG ACCATCC-3´ and TaqMan probe No. 42.

### Western Blot assay

The BMK-16/myc and SiHa cells were cultivated and treated with 100 ng/ml of ovine IFN-τ for 15 min. Then the cells were lysed with cold RIPA lysis buffer (Santa Cruz Biotechnology) with protease inhibitor cocktail (Sigma Aldrich) by incubating for 30 min at 4ºC, the total proteins were quantified using BCA protein assay kit (Price Rockford, il., USA) according to the manufacturer´s instructions. Fifty micrograms of total protein were separated by SDS-polyacrylamide gel electrophoresis 10% and transferred to a nitrocellulose membrane (Amersham Biosciences, Piscataway, NJ). Membranes were blocked with Tris-buffers saline (TBS) containing 0.5% Tween 20 and Blotto, no-fat dry milk (Santa Cruz Biotechnology) and the membrane were incubated with specific antibodies followed by horseradish peroxidase-conjugated secondary antibody incubation. The protein bands were detected using Price ECL Western Blotting substrate (Thermo Scientific). The antibody dilutions used were anti-p-JAK1 (Tyr 1022/Tyr 1023): sc-16773 (dilution 1:200), anti-JAK1 sc:295 (dilution 1:300) 130 kDa, anti-p-STAT1/Tyr-701) sc:7958 (dilution 1:300), anti-STAT1 sc-417 C-11 sc-417, anti-p-TyK2 (Tyr-1054/1055) sc-11763, anti-TyK2 sc-169 130kDa, anti-β actin C-11 sc-1615 (dilution 1:100) 43 kDa (Santa Cruz, Calif., USA).

### Statistical Analysis

Data were analyzed with the GraphPad Prism 5 software. For the JAK-STAT profiling, two-side unpaired Student's *t-*tests were used on the mean values from each experiment (IFN-τ-treated BMK-16/myc cells and non-treated cells for each protein). For the gene expression of the MHC class I, IP10 and MX2 an ANOVA test was performed. In both cases, p < 0.05 was considered statistically significant.

## Results

### Microarray Analysis of JAK-STAT Pathways in IFN-τ-Treated BMK-16/myc Cells

**Figure 1:**
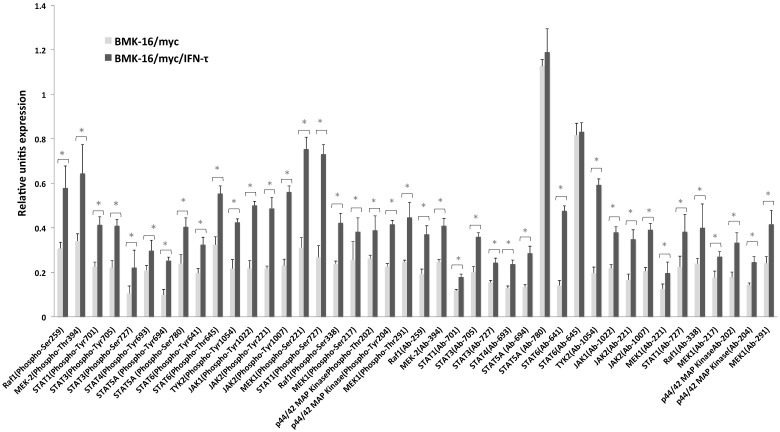
Antibody array data for JAK-STAT-proteins detection in BMK-16/myc cells treated with IFN-τ. In the experimental group, the phosphorylated and non-phosphorylated signal ratio induction was calculated in quadruplicate samples and the average was normalized with that of the *GAPDH* signal. The analysis of the JAK-STAT-protein detection was done comparing the BMK-16/myc cells treated with IFN-τ with the control group (without IFN-τ treatment). The statistical analysis shows the levels of phosphorylated and non-phosphorylated proteins of the JAK/STAT pathway by means a Two-side unpaired Students *t-*test were used on mean values from each experiment, with a significance of *p<0.05.

**Figure 2:**
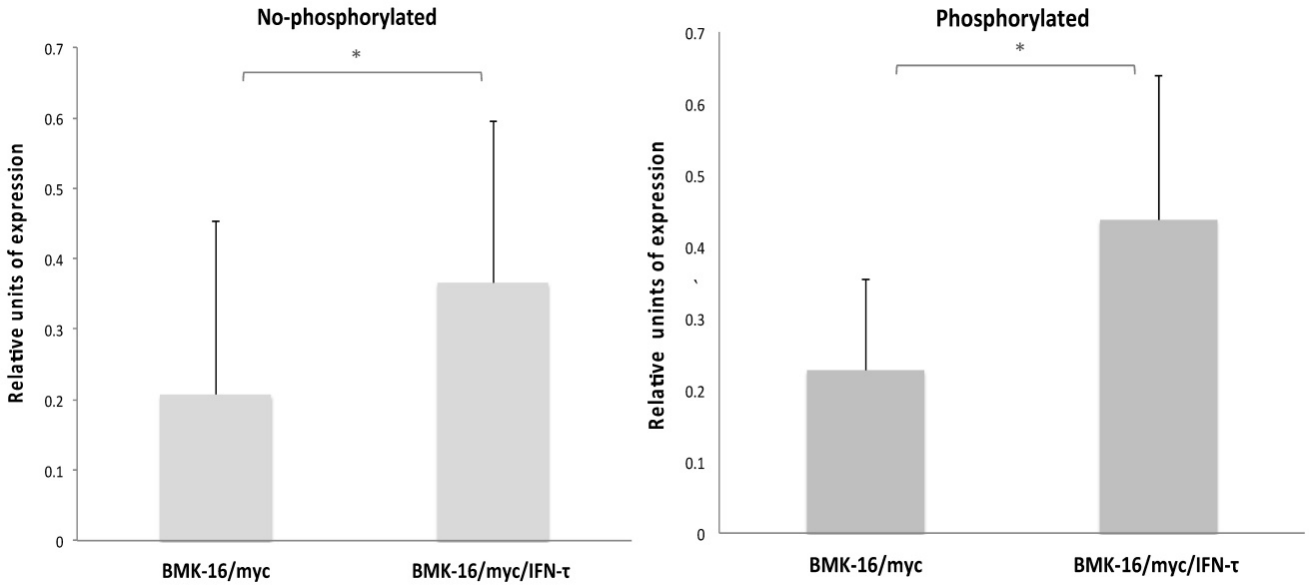
Ratio of JAK/STAT pathway levels phosphorylated and non-phosphorylated in BMK-16/myc cells treated with IFN-τ. **(A)** Non-phosphorylated profile of the JAK/STAT pathway significantly increased in the BMK-16/myc cell after they were treated exogenous IFN-τ. **(B)** Significant increased of ration JAK/STAT phosphorylated was detected in BMK-16/myc cell by action of IFN-τ. Data are represented as mean*SEM and two-side unpaired Student`s *t-*test were used on mean values from each gene expression vs control, with a significance of *p<0.05.

In order to determine which downstream signaling molecules are involved in the IFN-τ-mediated activation of the type-I interferon receptor in the HPV 16-transformed epithelial cells, we analyzed the activation of the phosphorylated and non-phosphorylated JAK-STAT pathways in BMK-16/myc cells treated with IFN-τ. The canonical signaling pathway was detected in the BMK-16/myc cells, JAK1, TYK2 and STAT1 proteins were identified in the cells without treatment with IFN-τ. However, after the treatment with IFN-τ, the detection of these proteins increased significantly (p < 0.05). These results were verified by the detection of proteins JAK1, TYK2, STAT1 by western blot in BMK-16/myc cells and SiHa cells by effect of IFN-τ (Supplementary Figure 1). Interestingly, proteins of the non-canonical signaling pathway were detected (MAPK cascade, MEK1, MEK2, Raf1, STAT3, STA4, STAT5 and STAT6), and it was determined that they were activated with IFN-τ (**Figure 1**). Moreover, IFN-τ favors the phosphorylation of the proteins from the canonical and non-canonical signaling pathways. JAK1 (Tyr1022), TYK2 (Tyr1054), and STAT1 (Tyr701) were detected, and it was determined that they were over activated by IFN-τ. Furthermore, the phosphorylated forms of MAPK kinase (JAK2, MEK1, MEK2, Raf1, STAT5 and SAT6) were significantly detected, and it was determined that they were activated by IFN-τ in the BMK-16/myc cell line. Furthermore, we analyzed the ratio of the protein levels between the phosphorylated and non-phosphorylated canonical and non-canonical-signaling pathways in the IFN-τ-treated BMK-16/myc cells (**Figure 2**). An increase in the non-phosphorylated protein profile was detected after the treatment with IFN-τ. However, the increase observed in the phosphorylated protein profile after the treatment with IFN-τ was much higher, in comparison with the non-treated cells.

### IFN-τ-Stimulated Genes IFN-τ in BMK-16/myc Cells

**Figure 3:**
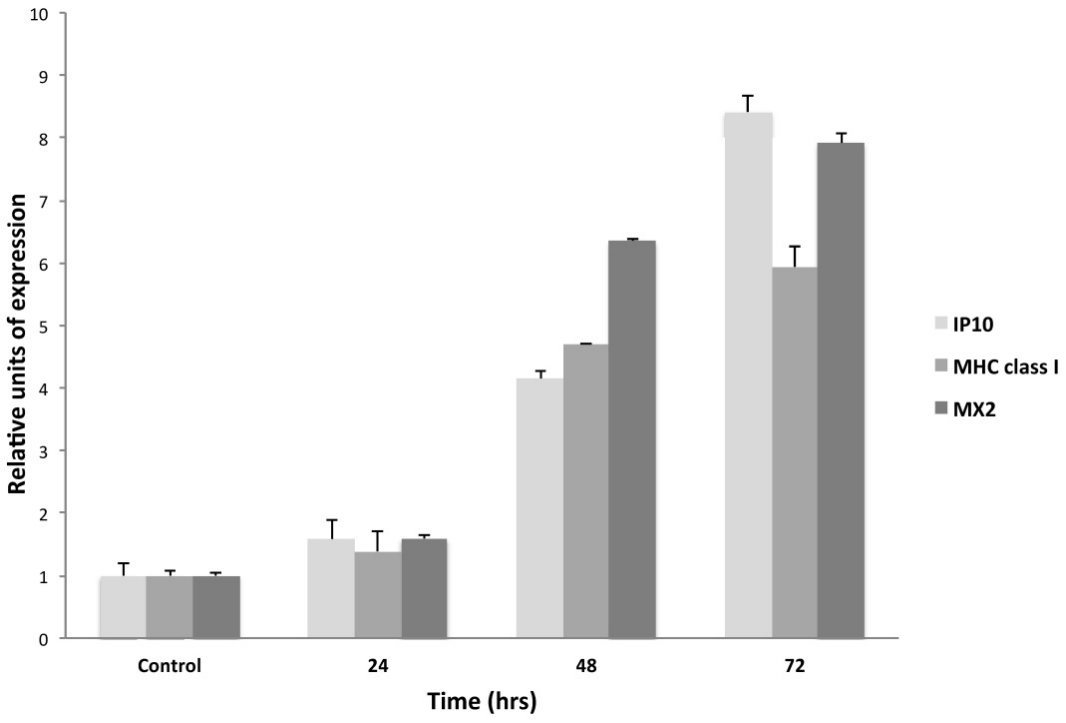
Relative expression levels of IFN-stimulated genes in BMK-16/myc cells treated with IFN-τ. The cells were treated with 100 ng IFN-τ for 72 h. Isolated total RNA was analyzed by real time RT-PCR to detect the expression of the IP10, MHC class I and MX2 genes with specific primers and specific TaqMan probes for the genes. The gene expression analysis was carried out with the comparative CT method through the formula 2^-∆∆Ct.^ Columns, the means of two experiments; bar, S.D. (*) Two-side unpaired Students *t-*test were used on mean values from each gene expression vs control, with a significance of *p<0.05.

In order to analyze the IFN-τ-stimulated genes in the BMK-16/myc cell line, cells were cultured and treated in presence or absence of IFN-τ (100 ng) for 24 and 48 hours. The expression of IP10, MHC class I and MX2 were detected by real time RT-PCR. These assays revealed that IFN-τ induces the expression of the IP10, MHC class I, and MX2. Forty-eight hours after the treatment, the cells showed a significant activation of the expression of the interferon-stimulated genes, and MX2 showed a slightly higher expression (**Figure 3**).

## Discussion

Recent finding showed that IFN-τ has antiviral activity and antiproliferative effects on tumor cells. It also displays a cross-species activity in humans, mice and ruminants, and it lacks the associated toxicity of other interferons 14,15. The fact that exogenous ovine IFN-τ has antiviral and antiproliferative effects on HPV16-positive murine and human cells is also correlated to the tumor inhibition in a cervical cancer experimental model 14, and it is linked to the activity of IFN-τ on the permissibility of tumor cells and signal transduction. In order to determine which downstream signaling molecules are involved in the HPV16-transformed epithelial cells, we examined the effects of the ovine interferon on the JAK-STAT-protein profiling and on the phosphorylation profiling of BMK-16/myc cells.

In this study, we showed that the canonical JAK-STAT-1 signaling pathway was detected in BMK-16/myc cells and that it was up regulated by IFN-τ. The microarray analysis showed that Tyk2, Jack1, Jack2 and their phosphorylated forms are activated by IFN-τ in tumor cells. This results were verified by western blot analysis, observed that IFN-τ high the phosphorylated forms STAT-1, JAK1 and TYK2 protein in BMK-16/myc. Also effect of IFN-τ on activation of canonical JAK-STAT-1 signaling pathway was observed in SiHa cells (human cervical cancer cell HPV 16 positive) western blot (Supplementary Figure 1). These results indicate that the ovine IFN-τ has effects on murine and mouse cell lines transformed with HPV 16 which corroborates the cross-species activity IFN-τ and the ability of IFN-τ to activate the activation of canonical JAK-STAT-1 signaling pathway in both cell lines. Addition, our analysis also revealed the activation of STAT-1, STAT-3, STAT-4, STAT-5 and STAT-6, and that STAT-6 is predominantly activated by IFN-τ (**Figure 1**). The activation of a classical JAK-STAT signaling pathway by IFN-τ has been determined in bovine trophoblast cells, bovine endothelial epithelial cells 16, bovine endometrium cells, and human fibroblast cells 6, but it had not been described in tumor cells. Moreover, our data clearly show the activation of MAPK kinase by IFN-τ in BMK-16/myc cells, as is the case with non-classical type-I IFNs. However, it has been speculated that type-I IFNs can cooperate with multiples distinct signaling cascades to generate a response 9,17. Specifically, it has been described that the type-I IFN-dependent antiviral properties are associated with the activation of the MAP kinase signaling cascades and that the p38α MAP kinase pathway regulates the activation of downstream effectors that participate in the induction of IFN-depended gene transcription, mediating the IFN-response 18. Recently has been reported that the activation of MAP44 is involved in the chemotaxis, motility and metabolism in bovine trophoblast cells mediates by IFN-τ 18. In our study, we did not analyze the p38 MAP kinase in tumor cells, but we speculated that this pathway could be activated since we detected the proteins MAP44 and MAP42 activated in the BMK-16/myc cells treated with IFN-τ. Nevertheless, we must consider the fact that we worked with transformed tumor cells and many mechanisms can be deregulated in favor of transformation and immortalization process.

**Figure 4:**
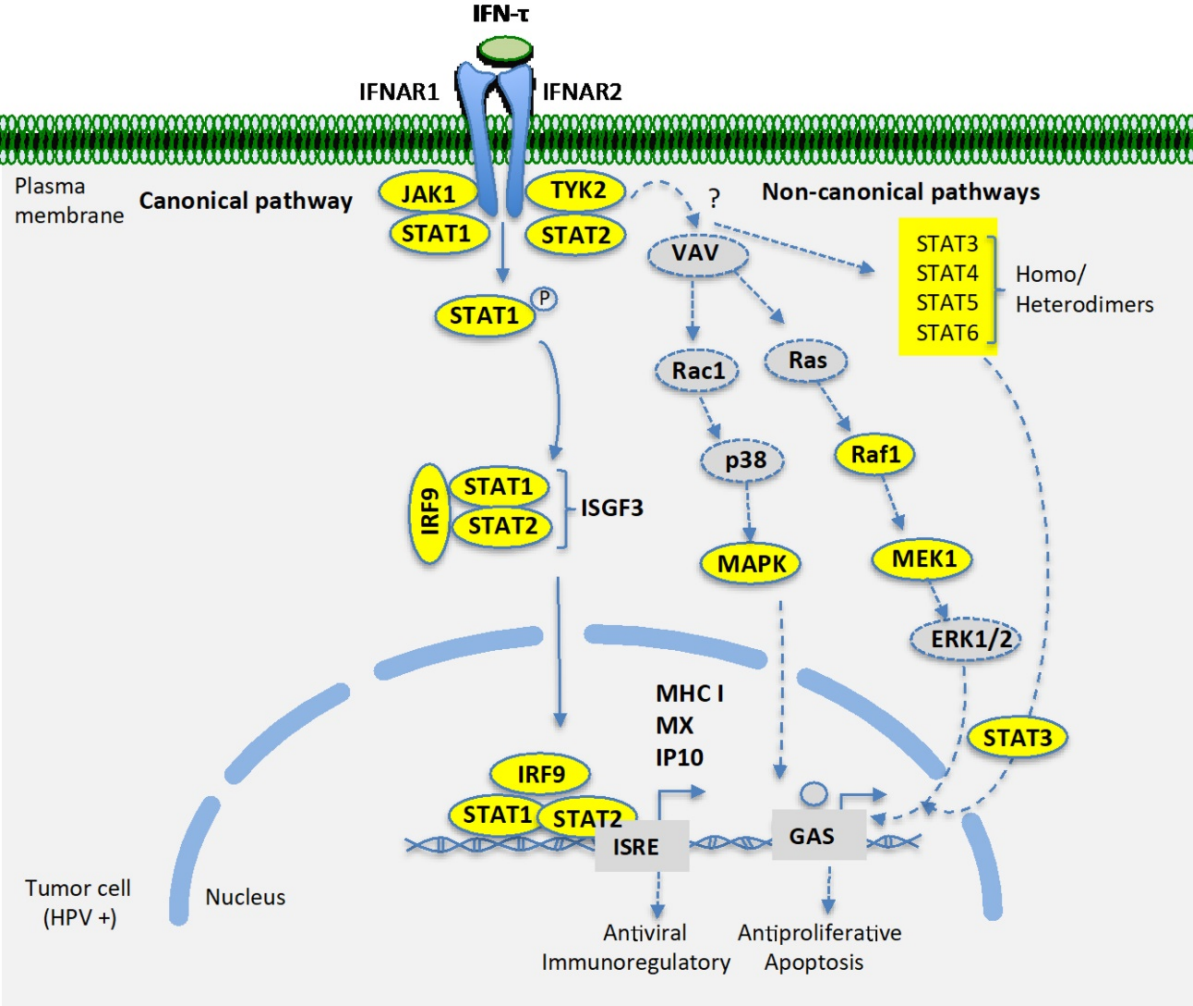
Schematic that illustrates the hypothesis of how the interferon tau (IFN-τ) acts on positive HPV 16 tumor cells. IFNτ binds to the receptor of the type I interferon family (rIFNs) and activates the JAK-STAT pathway in the tumoral cells which stimulates STAT1, STAT2 and resuts in the activation of the caconical pathway. However, other proteins that are involved in cell signaling corresponding to a non-canonical pathway are also detected. MAPK, MEK1, MEK2, Raf1, STAT3, STA4, STAT5 and STAT6 were observed, indicating the activation of other no-classical pathway HPV 16 tumor cells. These events may result in the induction of the expression of genes responding to type I interferon and to interferon-activated sites associated with the effects of antiviral, immunoregulatory, antiproliferative and apoptosis effects in tumor cells. IFNAR, Interferon-α/β receptor; STAT, Signal transducers and activators of transcription1, ISRE, Interferon-sensitive response element; GAS, Interferon-activated site; STAT1, Signal transducers and activators of transcription 1. IP10, Interferon-inducible protein 10; MX, myxovirus resistance protein; MHCI, Major histocompatibility complex; VAV, vav family proteins, MEK, Mitogen-activated proteine kinase; IRF9, Interferon regulatory factor 9; JAK, Janus kinase, TYK, Tyrosine kinase; ISGF3, Interferon stimulated gene factor.

Furthermore, it has been reported that IFN-τ induces the expression of classical type-I IFN-stimulated genes (ISGS) with a biological implication in conceptus elongations 20, but the biological role of IFN-τ in cancer cells has not been studied. Our results support the fact that IFN-τ induces the expression of the IP10, MHC classes I, and MX2 in BMK-16/myc cells. This indicates that the tumor cells allow the signaling transductions favored by IFN-τ and that IFN-τ exerts action on target genes that respond to type-I interferons. However, we can hypothesize that other ISGS may be induced by IFN-τ in tumor cells and that this may be associated to the inhibition of viral replication and to its antiproliferative action *in vitro* systems, as well as its immunoregulatory and antitumoral action in *in vivo* systems too (**Figure 4**). These results correlate with our previously stated findings 14, showing the therapeutic potential of interferon tau for the treatment of cervical cancer and its premalignant lesions.

In our investigation, a basic and fundamental question was to determine the presence of the signal transduction present in 16 HPV positive tumor cells stimulated with IFN-τ, to understand the mechanisms involved in the activation in tumor cell. The detection of activation of the canonical pathway in the tumor cells, which has direct implications on the cellular permissibility before interferon and in the biologic actions of IFN-τ. This classical pathway is widely described for IFN-α and β, which lead to the activation of genes such as MHC class I and MX2. However, a novel finding of a non-canonical alternative pathway partially detected in HPV16 positive tumor cells, which provides new evidence of the joint activation with the canonical pathway in tumor cells by IFN-τ. It has been described that the non-canonical signaling model should provide information on the regulation of cellular processes both homeostatic and non-homeostatic 21. Decades of research have shaped a complex network of IFN-JAK-SAT pathways, the appearance of functions canonical and canonical for the regulation and transcription of ISG, in cellular defense against the invasion of pathogens 22. Particularly for IFN-τ it has been described in which it can activate multiple STATs and has effects on ISRE, drive transcription genes in endothelial epithelial cells, and this type of transcription regulatory mechanism is novel and may be a unique effect of IFN-τ, because it has not been described for any type I or type II IFN 23. However it is necessary to deepen how the signaling network is structured in a cellular transformation event where there is a viral implication (HPV).

On the other hand, the ability of the ovine IFN-τ to induce the expression of MHC class I and MX2 in the HPV 16 positive tumor cells is very important. Previously we have shown that in cervical cancer mechanisms of evasion to the immune response associated with HPV infection. Among the mechanisms involved, the inhibition of MHC class I expression by viral proteins of HPV in biopsies of patients with cervical cancer is significantly described, leading to the inhibition of antigenic presentation by the cell infected with HPV. In this way the HPV-infected cell may not be recognized by the cytotoxic T cell and escapes immune surveillance. The recovery of MHC class I expression in tumor cells by IFN-τ would restore antigenic recognition by cytotoxic T cells to eliminate the tumor cell. Previously our group demonstrated that IFN-τ exerts anti-tumor effect in a murine model HPV 16 positive generated using the cell line BMK-16/myc used in this project, which supports this possibility. Likewise, the ability of IFN-τ to induce the expression of MX2 in BMK-16/myc cells, whose function is to inhibit viral replication. This function of IFN-τ is not new, previously it has been reported that IFN-τ stimulates the expression of ISG15, MX1 and MX2 in bovine uterine epithelial cells 24. The importance of induction of MX2 expression in BMK-16/myc by IFN-τ lies in the context of inhibition of viral replication, IFN-τ could inhibit HPV replication. This assertion is supported since it has been reported that IFN-τ has antiviral effects and exhibits potent suppression of human papollomavirus E6/E7 oncoprotein expression 25.

The results of this study indicate that the IFN-τ activate JAK-STAT pathway in HPV16-positive BMK-16/myc cell, canonical and non-canonical pathway was activated and results in MHC class I activation, MX and IP10 with implications antiviral and immunoregulatory. This is an original study focused on understanding the mechanisms involved in the response of tumor cells (HPV 16 positive) to an interferon that presents low toxicity. Which is presented the molecular bases to help explain how the IFN-τ acts in the signaling HPV-positive tumor cells and the therapeutic potential to cervical cancer treatment and eventually for other tumor cells.

## Supplementary Material

Supplementary figures and tables.
