# Supplementary material for: Bovine Interferon-Tau Activates Type I interferon-Associated Janus-signal Transducer in HPV16-positive Tumor Cell
Source: J Cancer. 2020 Jun 1;11(16):4754–61. doi: 10.7150/jca.33527 (PMC7330701; doi:10.7150/jca.33527)

Supplementary Figure 1:

Western Blot analysis of the JAK-STAT pathway in IFN-τ-sensituve tumor cells HPV 16 postives. BMk-16/myc and SiHa cell lines both HPV 16 tranformed were treated with IFN-τ (100 ng/ml) for 15 minutes. Fifty micrograms of proteins of cell lysates were used to examine the protein STAT1, JAK1 and TYK2, and phosphorylated form by Western blot analysis. Actin levels were used, as a control to make sure that equal amount of protein was present in the extracts.

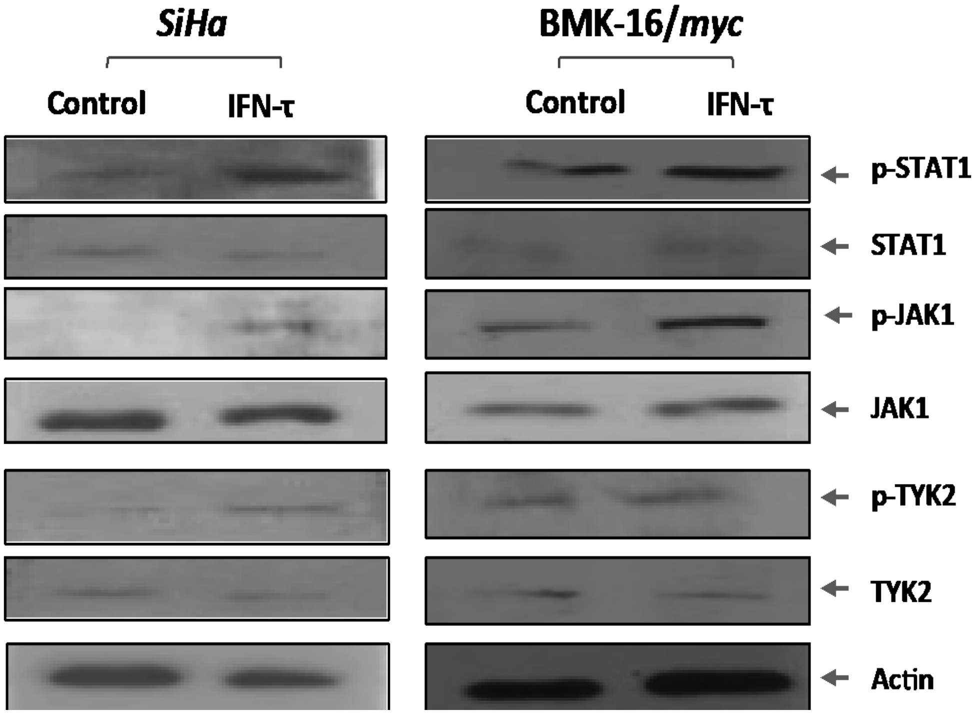

Supplement: Supplementary file 1 — Supplementary figures and tables. [file jcav11p4754s1.pdf]
